# Differential diagnosis of pulmonary *Talaromyces marneffei* infection and pulmonary tuberculosis in HIV/AIDS patients using a CT-based multimodal 2.5D deep learning approach

**DOI:** 10.3389/fcimb.2026.1927286

**Published:** 2026-10-23

**Authors:** Chang Song, Chun-Yan Zhao, Xiao-Shi Lin, Zhen-Tao Huang, Xue-Wen Huang, Shu-Lin Song, Zhou-Hua Xie, Qing-Dong Zhu

**Affiliations:** 1 Department of Tuberculosis, The Fourth People’s Hospital of Nanning, Nanning, Guangxi, China; 2 Clinical Medical School, Guangxi Medical University, Nanning, Guangxi, China; 3 Department of Radiology, The Fourth People’s Hospital of Nanning, Nanning, Guangxi, China

**Keywords:** 2.5D deep learning, HIV/AIDS, pulmonary tuberculosis, radiomics, *Talaromyces marneffei*

## Abstract

**Background:**

To investigate the diagnostic value of a computed tomography (CT)-based multimodal 2.5-dimensional (2.5D) deep learning model for differentiating Talaromyces marneffei (TSM) from pulmonary tuberculosis (PTB) in patients with human immunodeficiency virus infection/acquired immunodeficiency syndrome (HIV/AIDS).

**Methods:**

A total of 380 HIV/AIDS patients with pulmonary infections who were admitted to the Fourth People’s Hospital of Nanning from January 2018 to August 2025 were retrospectively enrolled, including 203 patients with PTB, 177 with TSM. The cohort was randomly divided into a training set and a test set at a ratio of 7:3. A clinical model, a radiomics model, and a 2.5D deep learning model were constructed separately.

**Results:**

In the clinical feature model, Random Forest achieved the optimal performance, with a test set AUC of 0.856. In the radiomics model, XGBoost attained the highest AUC of 0.866. In the 2.5D deep learning model, ResNet18 performed best at the slice level with an AUC of 0.865, and the patient-level support vector machine (SVM) model based on MIL achieved an AUC of 0.885. The multimodal combined model integrating clinical, radiomics, and deep learning features achieved the highest AUC of 0.931 and a specificity of 0.981. It significantly outperformed the clinical model (P = 0.020) and the radiomics model (P = 0.008), but the improvement over DL25D was not statistically significant (ΔAUC = 0.046; P = 0.116). The high specificity of the combined model should be interpreted alongside its sensitivity (0.717) and the selected decision threshold.

**Conclusion:**

The CT-based multimodal 2.5D deep learning model effectively integrates clinical indicators, radiomics features, and deep learning representations, and substantially improves the differentiation of TSM from PTB in HIV/AIDS patients.

## 1 Introduction

*Talaromyces marneffei* (TM), formerly known as *Penicillium marneffei*, is an endemic opportunistic dimorphic fungus that is primarily prevalent in Southeast Asian countries and Southern China (Supparatpinyo et al., 1994). This pathogen is highly virulent in individuals with severe immunosuppression and can cause Talaromyces marneffei (TSM) (Cao et al., 2019). Pulmonary tuberculosis (PTB) caused by *Mycobacterium tuberculosis* (MTB), also remains highly prevalent among patients with human immunodeficiency virus infection/acquired immunodeficiency syndrome (HIV/AIDS) (Getahun et al., 2010). Although these two pathogens differ in their manifestations of disease, their clinical manifestations, including prolonged fever, weight loss, cough, and lymphadenopathy, overlap substantially, early clinical differentiation difficult (Qiu et al., 2025; Zhao et al., 2025). Traditional differential diagnosis primarily relies on etiologic testing, including sputum smear microscopy, culture, and serologic assays (Zhao et al., 2025). Although these methods are regarded as diagnostic reference standards, they have limitations. First, culture is the gold standard for the diagnosis of TSM, but fungal growth may require up to 14 days, and can miss as many as 50% of infections, potentially delaying diagnosis and treatment initiation (Zhang et al., 2024). Microscopic examination of skin lesions, lymph nodes, or bone marrow, as well as histopathologic examination of tissue specimens, can provide a preliminary diagnosis; however, in patients without localized lesions, these methods are invasive, time-consuming, and impractical (Zhang et al., 2024). Second, profound immune dysfunction may reduce detection yield, and adequate deep respiratory specimens are often difficult to obtain without invasive procedures such as bronchoscopy or puncture biopsy. In clinical practice, physicians may therefore resort to empirical anti-tuberculosis or anti-fungal therapy before etiologic confirmation and infer the diagnosis from treatment response, an approach that inevitably increases diagnostic uncertainty and therapeutic risk. Accordingly, identifying deeper discriminative patterns from existing clinical and imaging data before etiologic confirmation has become an important research priority.

Imaging plays an important role in the screening and differential diagnosis of pulmonary opportunistic infections in patients with HIV/AIDS due to their non-invasiveness and wide accessibility (Spierling et al., 2023). Chest computed tomography (CT) can clearly display the morphology, distribution, boundaries, and anatomic relationshipa of pulmonary lesions, providing critical diagnostic clues. However, as mentioned above, the CT findings of TSM and PTB overlap considerably. Visual interpretation is highly dependent on radiologist experience and is difficult to standardize or quantify (Li et al., 2024; Wu et al., 2025; Niu et al., 2026). In recent years, with the rapid development of artificial intelligence (AI), deep learning (DL) has shown considerable promise in medical image analysis (Anderson et al., 2025). Deep convolutional neural networks (CNNs) can automatically learn high-dimensional representations from massive imaging data for esion detection, segmentation, and classification, and in some tasks their performance has approached or even exceeded that of human experts (Anderson et al., 2025). However, to date, no study has systematically explored the application value of a CT-based multimodal DL model in differentiating TSM from PTB in HIV/AIDS patients.

Against this background, this study aimed to construct a CT-based multimodal 2.5D DL model for the differential diagnosis of TSM and PTB in HIV/AIDS patients. This approach is expected to reduce misdiagnosis, shorten diagnostic delay, and improve clinical management, while also providing a new paradigm for the application of DL in the differential diagnosis of infectious pulmonary diseases.

## 2 Materials and methods

### 2.1 Subject sample inclusion

All consecutive patients admitted to our center between January 2018 and August 2025 who met the eligibility criteria were screened and enrolled retrospectively, with no intentional sampling or exclusion based on disease severity. Data were collected from hospitalized patients with HIV/AIDS at the Fourth People’s Hospital of Nanning between January 2018 and August 2025. The inclusion criteria were as follows: (1) HIV infection confirmed by an initial enzyme-linked immunosorbent assay (ELISA) screening test and confirmatory Western blot or immunofluorescence assay, or a documented diagnosis of AIDS; (2) availability of complete baseline clinical data, including age, gender, hospital admission number, major clinical symptoms (e.g., fever, cough, expectoration, dyspnea), and physical signs; (3) completion of high-resolution chest CT within 1 week before admission or before initiation of targeted anti-infective therapy after admission, with images covering the entire lung from apex to the base and meeting diagnostic quality requirements without severe respiratory motion or metal artifacts); (4) clear etiological confirmation; (5) age ≥ 18 years. The exclusion criteria were as follows: (1) simultaneous detection of TM and MTB; (2) coinfection with other opportunistic infections, such as Pneumocystis jirovecii pneumonia (PJP), cytomegalovirus (CMV) pneumonia, non-tuberculous mycobacterial (NTM) infection, cryptococcosis, or toxoplasmosis, confirmed by etiology or molecular methods; (3) coexistence of non-infectious pulmonary lesions, such as primary lung tumors, pulmonary metastases, or interstitial lung disease, that might interfere with imaging interpretation; (4) receipt of targeted anti-fungal therapy (e.g., amphotericin B, itraconazole, voriconazole) for more than 72 hours, or anti-tuberculosis treatment (isoniazid, rifampicin, ethambutol, pyrazinamide, etc.) for more than 72 hours before CT examination; (5) reconstruction slice thickness > 1 mm, or inability to perform isotropic 1-mm reconstruction; (6) missing key variables, such as CD4+ T lymphocyte count, etiological diagnostic evidence, or imaging reports, that could not be supplemented from the medical records; (7) pregnant or lactation; (8)immunosuppression attributable to causes other than HIV (e.g., chemotherapy, organ transplantation) were excluded to ensure a homogeneous HIV/AIDS study population; (9) ipsilateral lung surgery history. This study protocol was conducted in accordance with the Declaration of Helsinki and was approved by the Medical Ethics Committee of the Fourth People’s Hospital of Nanning (Ethics No [2025].52). Given the retrospective design and the anonymization of the data, the equirement for informed consent was waived. Baseline demographic characteristics and laboratory variables were systematically collected. Demographic data included age, gender, and lifestyle habits, such as smoking history, alcohol consumption. Clinical characteristics included hypertension, diabetes, and fever history. Laboratory variables included immunological markers (peripheral blood CD3, CD4, CD8 lymphocyte counts and their ratios), and blood routine and inflammatory markers, including white blood cell, red blood cell, hemoglobin, platelet counts; neutrophil, lymphocyte, monocyte, eosinophil, basophil differential counts; and C-reactive protein levels. Continuous variables were recorded as mean ± standard deviation, and categorical variables were presented as frequencies and percentages.

### 2.2 Diagnostic criteria

The diagnosis of Pulmonary Talaromyces marneffei infection (PTM) was established in accordance with the 2020 EORTC/MSGERC criteria, which emphasize the requirement of microbiological or histopathological evidence of pulmonary lesions. A confirmed diagnosis must meet one of the following conditions: (1) Diagnosis via respiratory specimens: Characteristic centrally septated yeast-like cells were detected via microscopic examination of sputum or bronchoalveolar lavage fluid (BALF), or TM was isolated through fungal culture (n=89); (2) Diagnosis via lung histopathology: Specimens obtained through percutaneous lung puncture or transbronchial lung biopsy showed histopathological features consistent with TM infection (such as intracellular yeast-like pathogens within macrophages), and the result was confirmed by culture or molecular biological assays (n=12); For patients with disseminated infection (positive blood culture, n=45; positive bone marrow aspiration, n=28; positive skin lesion examination, n=3), inclusion in the pulmonary infection study cohort required the simultaneous presence of abnormal pulmonary imaging findings (infiltrative lesions on chest CT, including ground-glass opacities, nodules, consolidation or cavities) and detection of the pathogen in respiratory specimens or lung tissue. Patients with only extrapulmonary dissemination and no evidence of pulmonary abnormalities on imaging or pathogen detection were excluded from this study (Cao et al., 2019; Donnelly et al., 2020). The diagnosis of PTB strictly followed the criteria issued by the Chinese Center for Disease Control and Prevention (WS 288-2017), and required MTB specific confirmation via at least one of the following approaches: (1) Positive result of *Mycobacterium tuberculosis* detected by Xpert MTB/RIF assay in sputum or bronchoalveolar lavage fluid (BALF) (n=98); (2) *Mycobacterium tuberculosis* isolated from sputum culture (n=76); or (3) Histopathological examination revealed caseating granuloma, combined with positive Xpert MTB/RIF result or positive culture from the same tissue specimen (n=29). Therefore, all included PTB cases were confirmed by culture or molecular assays.

### 2.3 CT image acquisition and parameters

For each patient, only one eligible chest CT examination was included. If the same patient was hospitalized multiple times or underwent chest CT multiple times, only the examination closest in time before initiating the targeted anti-infection treatment was included. All clinical variables, including complete blood count, CD4+ T lymphocyte count and C-reactive protein (CRP), were measured at baseline within 48 hours after admission, and before administration of any anti-tuberculosis or antifungal agents. Chest CT scans were performed within 1 week prior to admission or before initiation of anti-infective therapy. Patients who had received anti-tuberculosis or antifungal treatment prior to CT examination or blood collection were excluded, to ensure that all measurements reflected the state of untreated disease. The imaging data used in this study were derived from the standardized chest CT workflow of the hospital Department of Radiology. All examinations were performed on the same GE LightSpeed 64-slice spiral CT scanner to ensure consistency and comparability at the data acquisition stage. Before each examination, imaging technologists verified equipment operation and core scanning parameters in accordance with routine quality-control procedures to ensure stable image acquisition. Patients were positioned supine with both arms raised above the head to reduce obstruction of the lung field by the shoulder bones. Under standardized instruction, patients were asked to hold their breath at end-expiration during image acquisition to reduce respiratory motion artifacts and improve slice alignment. All scans were performed using a non-contrast helical protocol. The main technical parameters were as follows: slice thickness, 5 mm; tube voltage, 120 kV; and tube current modulation using automatic tube current modulation (ATCM). This protocol was used to balance radiation dose and image quality in accordance with the As Low As Reasonably Achievable (ALARA) principle. After raw data acquisition, image reconstruction was performed on the vendor workstation. To meet the needs of routine imaging interpretation and detailed lesion analysis, both standard and high-resolution reconstruction algorithms were applied. Standard reconstruction was used for overall assessment of the lungs and mediastinal structures, while the high-resolution algorithm enhanced the delineation of small lesions in the lung parenchyma, bronchial walls, and interlobular septa, thereby providing high-quality input for subsequent radiomics analysis and DL modeling. All eligible imaging data were exported in Digital Imaging and Communications in Medicine (DICOM) format and archived after quality control review. Before any slice extraction, data augmentation, feature selection, or model training, the entire patient cohort was randomly divided into training and test sets strictly at the patient level in a 7:3 ratio (training set: 266 patients; test set: 114 patients). All preprocessing steps, including image intensity normalization (Z-score), radiomic feature selection (mRMR and LASSO), and deep learning model optimization, were performed exclusively on the training set. The test set was used only once for final independent validation. Therefore, no slices from the same patient appeared in both the training and test sets.

### 2.4 ROI segmentation and feature extraction

For the exported DICOM chest CT images, standardized preprocessing was first performed using ITK-SNAP. Specifically, all images were resampled to a uniform voxel size of 1×1×1 mm³, and fixed window width and window level settings were applied to optimize visualization of lung parenchyma and lesion extent. After the raw data was acquired, true 1-mm slice thickness series were reconstructed from the original projection data on the manufacturer’s workstation, rather than obtaining thin slices by interpolating the existing 5-mm thick images. Both standard and high-resolution reconstruction algorithms were applied to this 1-mm dataset. Furthermore, the high-resolution reconstruction algorithm was specifically optimized to enhance the visualization of the lung parenchyma, bronchial walls, and interlobular septa, thereby providing high-quality inputs for subsequent radiomics analysis and deep learning modeling. The 1 mm isotropic voxels are critical for ensuring the reproducibility of radiomic features and the effective utilization of spatial information from adjacent slices in the 2.5D deep learning model. After preprocessing, two radiologists with at least five years of experience in thoracic imaging independently identified lesion boundaries and performed manual delineation in a blinded manner, without access to the patients’ clinical information or specific infection type. A Region of Interest (ROI) was delineated on each consecutive axial CT images, and the ROIs from all slices were then fused to construct a three-dimensional Volume of Interest (VOI) for radiomics analysis. Radiomics features were then extracted from the VOIs using the open-source PyRadiomics package (version 3.0). The extracted features covered three major categories: morphological features, first-order statistics features, and texture features. Morphological features quantified the 3D geometric properties of the lesion, including size, shape, volume, and spatial distribution. First-order statistics features described the distribution of voxel intensities within the VOI. Texture features quantified spatial heterogeneity and gray-level distribution patterns using the Gray Level Co-occurrence Matrix (GLCM), Gray Level Run Length Matrix (GLRLM), Gray Level Size Zone Matrix (GLSZM), and Neighboring Gray Tone Difference Matrix (NGTDM). To ensure reproducibility and stability, the Intraclass Correlation Coefficient (ICC) was used to evaluate the consistency of VOI delineation and extracted features between the two radiologists. Only features with CC value > 0.8 were retained for subsequent analysis. To optimize model performance and reduce overfitting, a hierarchical feature-selection strategy was applied. First, features not significantly associated with the target variable were removed using the unpaired t-tests or Mann-Whitney U tests (P < 0.05). Next, Pearson correlation coefficients were calculated to assess redundancy, and for feature pairs with an absolute correlation coefficient > 0.9, only the feature with stronger predictive power was retained. Subsequently, minimum Redundancy Maximum Relevance (mRMR) was introduced for further feature refinement, reducing the feature dimension to 32 variables. Finally, a least absolute shrinkage and selection operator (LASSO)-regularized logistic regression model with 10-fold cross-validation was used to select features with non-zero coefficients, yielding the final feature subset with the greatest discriminative value. To prevent data leakage, all feature selection procedures—including univariate filtering, correlation analysis, mRMR ranking, and LASSO regularization—were exclusively conducted on the training cohort (N = 266). The test cohort (N = 114) was strictly reserved for final model evaluation using features and coefficients derived solely from the training data.

### 2.5 Deep learning framework

#### 2.5.1 Slice-based data preparation

A 2.5D representation was constructed by selecting the axial slice with the largest tumor area and incorporating surrounding anatomical context. Specifically, seven slices per subject were extracted, comprising the central slice along with its immediate (± 1), moderate (± 2), and extended (± 4) neighboring slices. This design captures both detailed tumor appearance and broader spatial information. The 2.5D representation was constructed by selecting the axial slice with the largest cross-sectional area of the lesion. A square cropped region centered on the lesion was extracted from this central slice: the size of the region was defined to cover the maximum diameter of the lesion with an additional 10-pixel margin, which ensured complete coverage of the lesion while minimizing background noise. Subsequently, the same spatial cropping coordinates were applied to six adjacent slices (± 1, ± 2, ± 4 slices relative to the central slice) to maintain spatial correspondence in the slice stack.

#### 2.5.2 Slice-level model training

Each slice was assigned the patient-level label and treated as an independent training instance in a classification. To leverage prior visual knowledge, transfer learning was employed using ImageNet-pretrained convolutional neural networks as backbone architectures. All input channels were normalized via Z-score transformation. During training, data augmentation included random cropping and horizontal/vertical flipping; during inference, only normalization was applied. All slices were resized to 224×224 pixels, and intensity values were scaled to [−1, 1]. Training Details: Optimization was performed with stochastic gradient descent (SGD) under a cosine annealing learning rate schedule:


$${\text{η}}_{\text{t}}={\text{η}}_{\text{min}}^{\text{i}}+\frac{1}{2}({\text{η}}_{\text{max}}^{\text{i}}-{\text{η}}_{\text{min}}^{\text{i}})(1+\text{cos}(\frac{{\text{T}}_{\text{cur}}}{{\text{T}}_{\text{i}}}\text{π}))$$

where ${\text{η}}_{\text{min}}^{\text{i}}=0$, ${\text{η}}_{\text{max}}^{\text{i}}=0.01$, and ${\text{T}}_{\text{i}}=32$ denotes the total number of training epochs. The loss function was defined as softmax cross-entropy. Each slice was processed as an independent 2D input (224 × 224 × 3 channels) via the ResNet-18 backbone network to generate slice-level probability predictions. Notably, the proposed 2.5D architecture does not employ 3D convolution; instead, spatial context information is integrated a posteriori through the multi-instance learning (MIL) framework, which aggregates predictions from the ordered slice stack (± 1, ± 2, ± 4 slices relative to the central axial slice) to capture the spatial distribution of lesion features.

#### 2.5.3 Multi-instance learning for patient-level fusion

To aggregate slice-level predictions and integrate them with radiomic descriptors, a multi-instance learning (MIL) framework was adopted. Detailed procedures for the MIL framework are provided in **Supplementary Material 1A**. Two complementary strategies were implemented: the Predict Likelihood Histogram (PLH) approach and Bag of Words (BoW) approach. In the PLH approach, slice-level probabilities (Slice_prob) and class predictions (Slice_pred) generated by the 2.5D models were aggregated into histogram distributions. These histograms were normalized via min-max scaling to generate Histo_prob and Histo_pred feature vectors, which captured the overall predictive profile across slices. In the BoW approach, a dictionary of unique slice-level prediction patterns was first established. Each patient’s slices were then encoded according to the frequency of these patterns, and the resulting counts were weighted using Term Frequency–Inverse Document Frequency (TF–IDF) to generate Bow_prob and Bow_pred features. These features were intended to emphasize recurrent discriminative patterns across the slice set. The final fused representation was obtained by concatenating all four feature groups:


$${\text{feature}}_{\text{fusion}}={\text{Histo}}_{\text{prob}}⊕{\text{Histo}}_{\text{pred}}⊕{\text{Bow}}_{\text{prob}}⊕{\text{Bow}}_{\text{pred}}.$$

### 2.6 Model construction and evaluation

In all analyses, PTB was defined as the positive class and TSM as the negative class; accordingly, true positives (TP) and false negatives (FN) referred to PTB patients who were correctly and incorrectly classified, respectively, while true negatives (TN) and false positives (FP) referred to TSM patients who were correctly and incorrectly classified, respectively. All univariable and multivariable logistic regression analyses were performed exclusively in the training cohort (N = 266; 143 patients with PTB and 123 with TSM), while the test cohort (N = 114) was used only for final model evaluation and did not contribute to any feature selection or regression modeling. Each patient contributed only one observation; no repeated observations, weighting, or synthetic samples were used, and SMOTE was not applied to the clinical regression data. The dependent variable was coded as PTB = 1 and TSM = 0. Binary variables, such as fever, diabetes, and hypertension, were coded as 0/1, whereas continuous variables, such as CD4+ T lymphocyte count, platelet count, hemoglobin, and basophils, were entered into the models using their original values. Missing data were handled by complete-case analysis without imputation. The selected features were used to train both linear and non-linear classifiers. Linear modeling was performed using Support Vector Machines (SVM), while non-linear relationships were captured using Random Forest (RF), Decision Trees, XGBoost, and LightGBM. Hyperparameters for all classifiers were optimized using a combination of grid search and 5-fold cross-validation. To ensure conceptual clarity regarding the hierarchical architecture of the proposed framework, it is essential to delineate the relationship between the 2.5D deep learning pipeline, the MIL-based aggregation, and the final multimodal integration. Specifically, the 2.5D ResNet-18 serves as a slice-level feature extractor, generating probabilistic predictions for seven adjacent CT slices centered on the maximal lesion cross-section. These slice-level outputs are subsequently aggregated via the MIL framework (encompassing PLH and BoW strategies) into a patient-level discriminative signature designated as DL25D. This DL25D signature represents the deep learning modality within the multimodal framework. The final combined model is constructed through early fusion at the feature level, wherein the DL25D signature is concatenated with radiomic features (extracted from three-dimensional VOIs) and clinical variables (selected via multivariate logistic regression), thereby integrating learned hierarchical imaging representations with handcrafted quantitative features and curated domain knowledge for robust differential diagnosis. For comparative evaluation, the proposed MIL-based fusion framework was compared with traditional ensemble fusion methods, additional implementation details are provided in **Supplementary Material 1B**. An independent clinical feature set was constructed by retaining only variables that were statistical significance in both univariate and multivariate logistic regression analyses. To fully utilize the complementary information across modalities, a comprehensive predictive feature set was then created by concatenating the discriminative features or high-level representations derived from the radiomics model, the 2.5D DL model, and the clinical feature set. The final fused feature vector, obtained by concatenating clinical, radiomics, and 2.5D DL features, was fed into a Support Vector Machine (SVM) classifier with a linear kernel, which was chosen for its superior generalisation performance observed in the patient-level MIL evaluation. To convert SVM decision scores to clinically interpretable probabilities, we applied Platt scaling (logistic regression on the SVM decision function) using scikit-learn’s CalibratedClassifierCV with cv=‘prefit’. Critically, the calibration function was fitted exclusively on out-of-fold predictions generated from the training cohort during the nested cross-validation procedure, ensuring no test set information influenced the calibration mapping. The final probability estimates for the test set were derived by applying this training-set-fitted calibration function to the locked test set SVM scores. Model selection was based on a comprehensive evaluation of multiple performance metrics and generalization ability within the training set, rather than on test-set performance. Using patient-level nested cross-validation, candidate models were compared according to the mean AUC across the outer validation folds, sensitivity, specificity, PPV, NPV, calibration, and the gap between training-fold and validation-fold performance. Models showing a favorable balance across these metrics and a smaller training-to-validation gap were considered to have better generalization ability and were preferentially selected. All preprocessing, feature selection, SMOTE, BoW dictionary construction, and hyperparameter tuning were performed exclusively on the outer training folds. The test set was locked and used only once for final evaluation, and did not influence any modeling decision. For example, the reported training-set AUC of 0.901 and specificity of 0.943 for XGBoost, its decrease to a test-set AUC of 0.847, and the highest test-set AUC of 0.856 for RF are *post hoc* comparative reports after model determination and were not used to retrospectively change features, model architecture, fusion strategy, or hyperparameters. Model performance was evaluated using multiple metrics. Discrimination was measured by the Area Under the Receiver Operating Characteristic Curve (AUC). Calibration was assessed by calibration curves and the Hosmer-Lemeshow test. Clinical utility was further quantified by Decision Curve Analysis (DCA) to estimate the net benefit at different threshold probabilities.

### 2.7 Statistical analysis

Normality of continuous variables was assessed using the Shapiro–Wilk test. Depending on data distribution, continuous variables were compared using Student’s t-test or the Mann–Whitney U test, while categorical variables were compared using the chi-square test. Baseline characteristics were generally balanced between cohorts, with no statistically significant differences observed (all p > 0.05). All analyses were performed in Python 3.7.12. Statistical analysis was conducted using Statsmodels 0.13.2. The 95% confidence intervals for AUCs were estimated using 1,000 bootstrap resamples, with the 2.5th and 97.5th percentiles of the bootstrap distribution reported as the confidence interval. Radiomics feature extraction was performed using PyRadiomics 3.0.1, machine learning models were implemented using Scikit-learn 1.0.2, and DL models were constructed using the PyTorch 1.11.0 with CUDA 11.3.1 and cuDNN 8.2.1 acceleration.

## 3 Results

### 3.1 Clinical model

According to the inclusion and exclusion criteria, a total of 380 patients were enrolled in this study, comprising 203 patients with PTB and 177 with TSM (**Figure 1**). Baseline characteristics differed significantly between groups (**Supplementary Table 1**). TSM patients exhibited lower CD4+ T lymphocyte counts (median 17 vs. 127 cells/μL), platelet counts (137 vs. 219 ×10^9^/L), and hemoglobin (81 vs. 102 g/L) compared to PTB (all p<0.001). Conversely, PTB patients were older with higher hypertension and diabetes prevalence. These distinct host immune and comorbidity profiles support integrating clinical variables with imaging features for differential diagnosis. Eligible cases were randomly assigned to the training and test sets at a ratio of 7:3. Baseline characteristics are presented in **Supplementary Table 2**. No statistically significant differences were observed between the two groups in age, cellular immunity (CD3, CD4, CD8), routine hematologic parameters (including white blood cells and platelet counts), or common comorbidities such as hypertension, diabetes, suggesting good comparability between groups. The cohort consisted of 296 males (77.9%) and 84 females (22.1%), resulting in a male-to-female ratio of approximately 3.5:1, which is consistent with the epidemiological characteristics of the HIV/AIDS population in southern China, where males predominate. In the training set ( n=266), there were 207 males (77.82%) and 59 females (22.18%), while the test set ( n=114) comprised 89 males (78.07%) and 25 females (21.93%). There was no statistically significant difference in gender distribution between the two groups (χ² test, P=1.000), indicating that the random split achieved an ideal balance in terms of gender. Univariate analysis indicated that fever, CRP, platelet count, lymphocyte count, and diabetes mellitus were significantly associated with the outcome. Multivariate analysis further identified fever, CD4+ T lymphocyte count, platelet count, hemoglobin level, diabetes, and hypertension as independent predictors (**Supplementary Table 3**). Based on these clinical features, five machine learning algorithm models were constructed and compared (**Table 1**). The RF model showed the best generalization performance, achieving the highest AUC (0.856) in the test set while maintaining a favorable balance between sensitivity (0.817) and specificity (0.815). All univariate and multivariate logistic regression analyses for clinical feature selection were performed exclusively on the training set (N = 266). The identified independent predictors were subsequently used to construct models on the training set, with performance evaluated on the held-out test set (N = 114).

**Figure 1:**
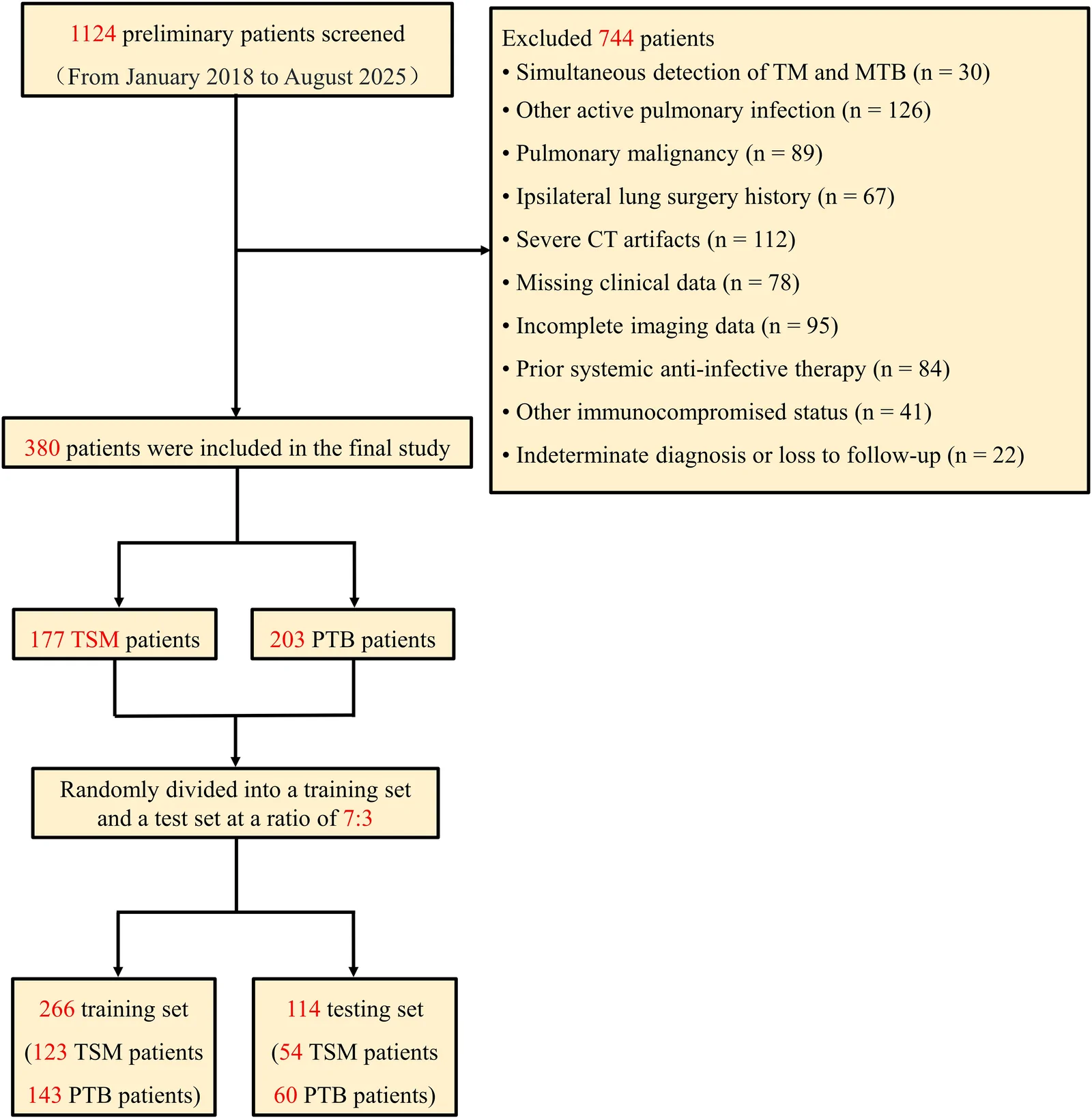
Patient inclusion flowchart.

**Table 1:** Clinical model performance of different machine learning algorithms.

Model name	AUC	95% CI	Sensitivity	Specificity	PPV	NPV	Cohort
SVM	0.818	0.767~0.869	0.755	0.821	0.831	0.743	train
SVM	0.811	0.732~0.890	0.667	0.889	0.870	0.706	test
RandomForest	0.881	0.841~0.920	0.762	0.862	0.865	0.757	train
RandomForest	0.856	0.786~0.925	0.817	0.815	0.831	0.800	test
ExtraTrees	0.838	0.792~0.884	0.692	0.813	0.811	0.694	train
ExtraTrees	0.832	0.757~0.908	0.733	0.907	0.898	0.754	test
XGBoost	0.901	0.866~0.937	0.734	0.943	0.937	0.753	train
XGBoost	0.847	0.778~0.916	0.700	0.833	0.824	0.714	test
LightGBM	0.869	0.828~0.910	0.825	0.732	0.781	0.783	train
LightGBM	0.845	0.776~0.914	0.667	0.907	0.889	0.710	test

### 3.2 Radiomics model

A total of 1,561 quantitative radiomics features were extracted from the ROIs using the PyRadiomics platform, comprehensively characterizing lesion gray-level distribution, morphological properties, and textural heterogeneity. To reduce dimensionality and select the most discriminative variables, LASSO regression with 10-fold cross-validation was introduced. Ultimately, 11 features with non-zero coefficients were selected as the core variables for model construction. Details regarding the selection process and coefficient distribution are provided in **Supplementary Figures 1A–C**. The predictive performance of five machine learning algorithms was then evaluated in the training and test sets (**Table 2**). XGBoost demonstrated the best generalization performance, with the smallest difference between the training and test set AUCs (0.873 vs. 0.866).

**Table 2:** Radiomics model performance of different machine learning algorithms.

Model name	AUC	95% CI	Sensitivity	Specificity	PPV	NPV	Cohort
SVM	0.855	0.809~0.901	0.902	0.715	0.787	0.863	train
SVM	0.851	0.780~0.922	0.817	0.815	0.831	0.800	test
RandomForest	0.873	0.830~0.916	0.825	0.829	0.849	0.803	train
RandomForest	0.831	0.753~0.909	0.717	0.852	0.843	0.730	test
ExtraTrees	0.823	0.773~0.873	0.741	0.789	0.803	0.724	train
ExtraTrees	0.812	0.731~0.892	0.700	0.815	0.808	0.710	test
XGBoost	0.873	0.834~0.913	0.657	0.902	0.887	0.694	train
XGBoost	0.866	0.798~0.934	0.883	0.722	0.779	0.848	test
LightGBM	0.871	0.830~0.912	0.776	0.829	0.841	0.761	train
LightGBM	0.810	0.730~0.889	0.883	0.630	0.726	0.829	test

### 3.3 Results of slice level model

The performance of various deep neural network architectures was compared in the training and test sets (**Table 3**). In the test set, ResNet18 performed the best overall performance, with AUC of 0.865, specificity of 0.918, and PPV of 0.892, all of which superior to those of other architectures. Furthermore, the difference in performance between the training and test sets was relatively small, demonstrating better generalization. Although ResNet50 achieved a high AUC of 0.937 in the training set, its AUC decreased substantially 0.782 in the test set, indicating a risk of overfitting. The Densenet models were also performed less well than ResNet18 overall, with DenseNet121 showing a specificity of only 0.534. These results indicate that, for the image-classification task in this study, ResNet18 provided a favorable balance between computational efficiency and discriminative performance, supporting its use as the backbone network for subsequent multimodal fusion.

**Table 3:** Metrics on slice level predictions.

Model name	AUC	95% CI	Sensitivity	Specificity	PPV	NPV	Cohort
Resnet18	0.884	0.8698~0.8992	0.797	0.804	0.825	0.773	train
Resnet18	0.865	0.8418~0.8890	0.607	0.918	0.892	0.678	test
Resnet50	0.937	0.9272~0.9475	0.830	0.892	0.899	0.819	train
Resnet50	0.782	0.7509~0.8139	0.776	0.667	0.721	0.728	test
Resnet101	0.821	0.8027~0.8402	0.757	0.744	0.775	0.725	train
Resnet101	0.754	0.7210~0.7873	0.605	0.788	0.760	0.642	test
Densenet121	0.847	0.8303~0.8645	0.797	0.739	0.780	0.758	train
Densenet121	0.730	0.6959~0.7643	0.807	0.534	0.658	0.714	test
Densenet201	0.925	0.9140~0.9365	0.840	0.843	0.862	0.819	train
Densenet201	0.792	0.7612~0.8225	0.771	0.693	0.736	0.732	test

### 3.4 Grad-CAM

To explore the spatial basis of model predictions, we applied Grad-CAM to visualize activation patterns in the final convolutional layer. As shown in **Figure 2**, the activation maps for two representative true-positive cases (one PTB, one TSM) demonstrate that the model focuses on regions containing the pulmonary lesions rather than background anatomy. In the PTB case, activation is concentrated within and around the cavitary lesion; in the TSM case, activation overlies the nodular infiltrates. These visualizations suggest that the model attends to clinically relevant image regions, though they do not demonstrate recognition of specific pathological features such as cavity-wall irregularity or satellite nodules.

**Figure 2:**
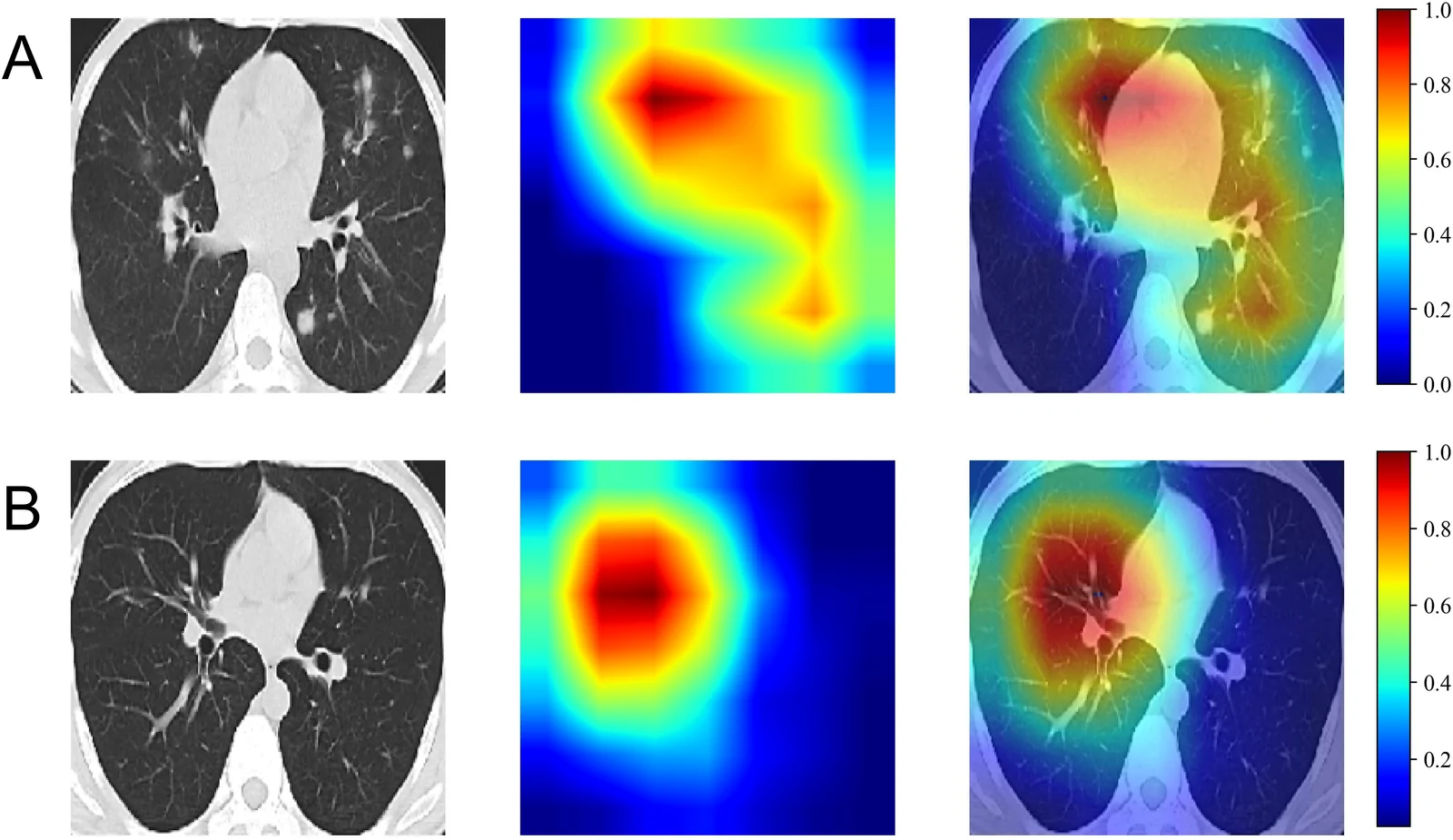
Grad-CAM visualizations of representative cases. (**(A)** A 34-year-old male with PTB; **(B)** A 41-year-old male with TSM).

### 3.5 Results of patient level model

Among the various machine learning models evaluated at the patient level, SVM exhibited the best generalization performance, with a test set AUC of 0.885, outperforming other algorithms (**Table 4**). Although ensemble models such as RF and XGBoost achieved near-perfect performance in the training set (AUC = 0.987), their performance declined substantially in the test set, suggesting overfitting. In contrast, SVM showed the smallest discrepancy between the training and test AUCs (0.944 vs. 0.885) and achieved a good balance between specificity (0.926) and sensitivity (0.667).

**Table 4:** Performance metrics of the multi-instance learning (MIL)-based prediction models.

Model name	AUC	95% CI	Sensitivity	Specificity	PPV	NPV	Cohort
SVM	0.944	0.917~0.970	0.853	0.927	0.931	0.844	train
SVM	0.885	0.829~0.942	0.667	0.926	0.909	0.714	test
RandomForest	0.960	0.938~0.982	0.937	0.959	0.964	0.929	train
RandomForest	0.845	0.772~0.917	0.667	0.907	0.889	0.710	test
ExtraTrees	0.877	0.846~0.908	0.594	0.992	0.988	0.678	train
ExtraTrees	0.827	0.755~0.900	0.633	0.963	0.950	0.703	test
XGBoost	0.987	0.974~0.999	0.965	0.976	0.979	0.960	train
XGBoost	0.856	0.790~0.923	0.717	0.870	0.860	0.734	test
LightGBM	0.962	0.941~0.984	0.874	0.976	0.977	0.870	train
LightGBM	0.849	0.784~0.914	0.667	0.907	0.889	0.710	test

### 3.6 Comparison of signatures

This study compared the performance of four prediction models: the clinical feature model, the radiomics model, the 2.5D deep learning model (DL25D), and the multimodal combined model integrating all three models (**Table 5**). In the test set, DL25D outperformed the radiomics model, achieving an AUC of 0.885, with specificity and PPV of 0.926 and 0.909, respectively. The combined model, which further integrated multimodal information, yielded the optimal performance, with an AUC of 0.931, and a specificity of 0.981 (**Figures 3A, B**). This represented a clear improvement over the single DL25D model. These results indicate that the multimodal fusion strategy effectively integrates deep DL-derived and handcrafted features, significantly enhancing discriminative performance and potential clinical utility.

**Table 5:** Prediction performance of different models.

Signature	TP	FN	TN	FP	AUC	95% CI	Sensitivity	Specificity	PPV	NPV	Cohort
Clinical	109	34	106	17	0.881	0.841~0.9200	0.762	0.862	0.865	0.757	train
Radiomics	94	49	111	12	0.873	0.834~0.9132	0.657	0.902	0.887	0.694	train
DL25D	122	21	114	9	0.944	0.917~0.9701	0.853	0.927	0.931	0.844	train
Combined	133	10	111	12	0.963	0.943~0.9839	0.930	0.902	0.917	0.917	train
Clinical	49	11	44	10	0.856	0.787~0.9247	0.817	0.815	0.831	0.800	test
Radiomics	53	7	39	15	0.866	0.798~0.9341	0.883	0.722	0.779	0.848	test
DL25D	40	20	49	4	0.885	0.829~0.9423	0.667	0.926	0.909	0.714	test
Combined	43	17	53	1	0.931	0.888~0.9753	0.717	0.981	0.977	0.757	test

The combined model employs feature concatenation followed by SVM classification.

**Figure 3:**
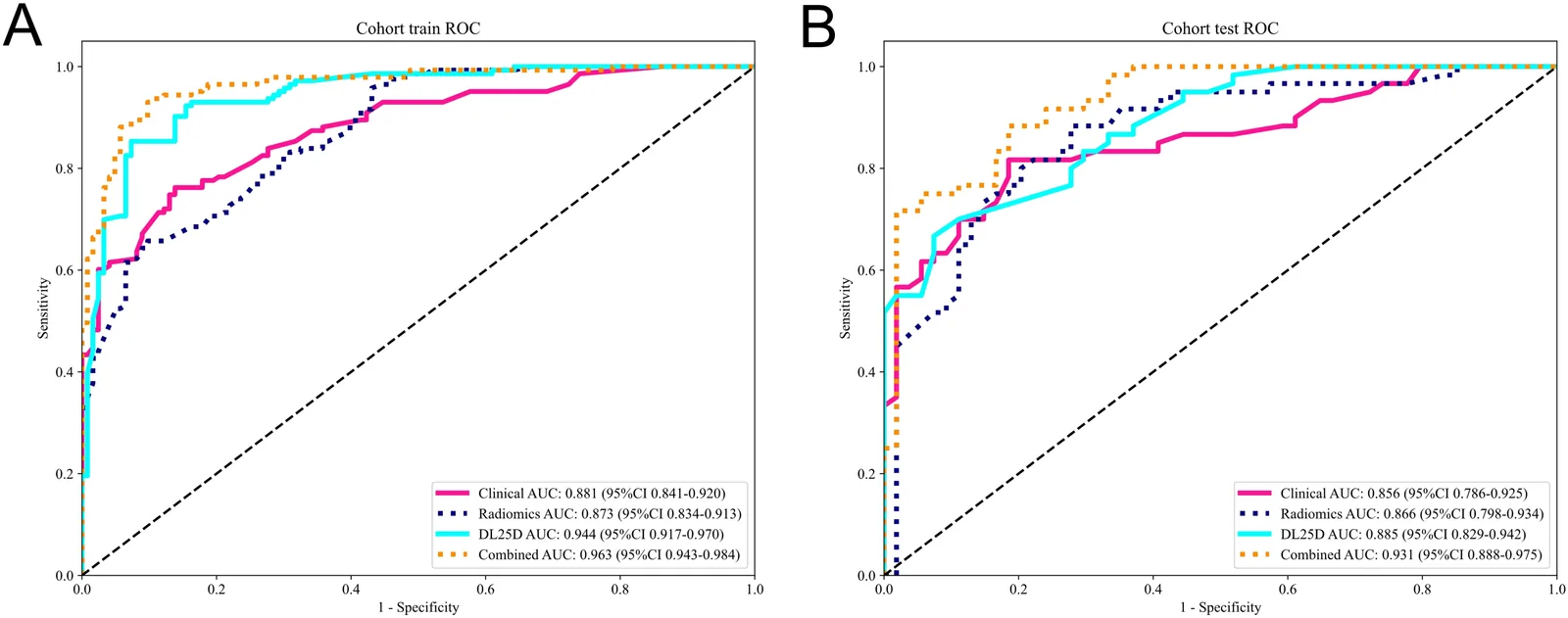
ROC comparison of clinical, radiomics, and fusion models. (**(A)** Training cohort; **(B)** Testing cohort).

### 3.7 Model performance evaluation and clinical application

Model calibration was evaluated using the Hosmer-Lemeshow goodness-of-fit test. The combined model exhibited excellent calibration in both the training and test sets, with Hosmer-Lemeshow test P values of 0.855 and 0.266, respectively, indicating good consistency between the predicted probabilities and actual clinical outcomes. In the training set, calibration intercepts ranged from −0.05 to −0.08 and slopes from 0.88 to 0.92, while Brier scores ranged from 0.076 (Combined) to 0.142 (Clinical). In the test set, intercepts ranged from −0.12 to −0.18 and slopes from 0.72 to 0.78; the Combined model achieved the lowest Brier score (0.089), followed by DL25D (0.112), Radiomics (0.149), and Clinical (0.156). These findings indicate that the Combined model had the best overall calibration and probabilistic accuracy (**Figures 4A, B**). Furthermore, the DeLong test was used to compare AUCs among models. The combined model demonstrated significantly superior discriminative performance compared to the Clinical model (ΔAUC = 0.075, 95% CI: 0.012–0.138, P = 0.020) and the radiomics model (ΔAUC = 0.065, 95% CI: 0.018–0.112, P = 0.008). However, the improvement over the DL25D model did not reach statistical significance (ΔAUC = 0.046, 95% CI: -0.012–0.104, P = 0.116), suggesting that while multimodal fusion shows promise, the incremental value over deep learning alone requires confirmation in larger cohorts (**Figures 4C, D**). As shown in **Figure 5**, the Combined model provided the highest net benefit across a broad threshold range of approximately 0.10–0.90 in both the training and test cohorts, outperforming the clinical, radiomics, and DL25D models as well as the treat-all and treat-none strategies. In the test cohort, this advantage persisted from 0.10 to 0.90 and declined only at extremely high thresholds (>0.95), where very few patients would be classified as positive. A threshold range of 0.20–0.80 may be clinically most relevant, with lower thresholds favoring sensitivity to avoid missing TSM and higher thresholds favoring specificity to avoid unnecessary antifungal therapy. Compared with the clinical model, the combined model also yielded higher net benefit across this range.

**Figure 4:**
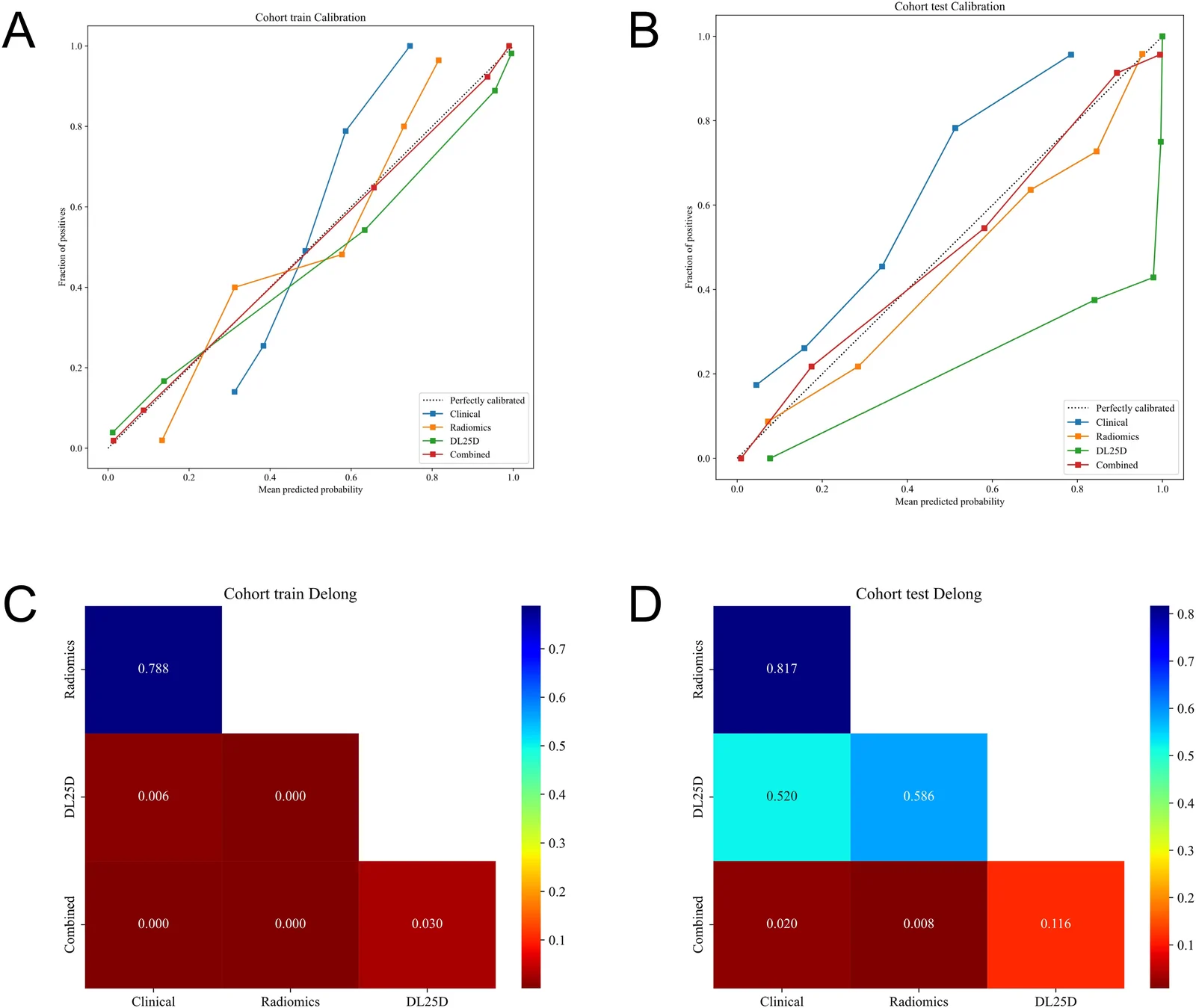
Model calibration and statistical performance comparison. (**(A)** Calibration curve of the training cohort; **(B)** Calibration curve of the testing cohort; **(C)** P-value matrix of Delong test for the training cohort; **(D)** P-value matrix of Delong test for the testing cohort).

**Figure 5:**
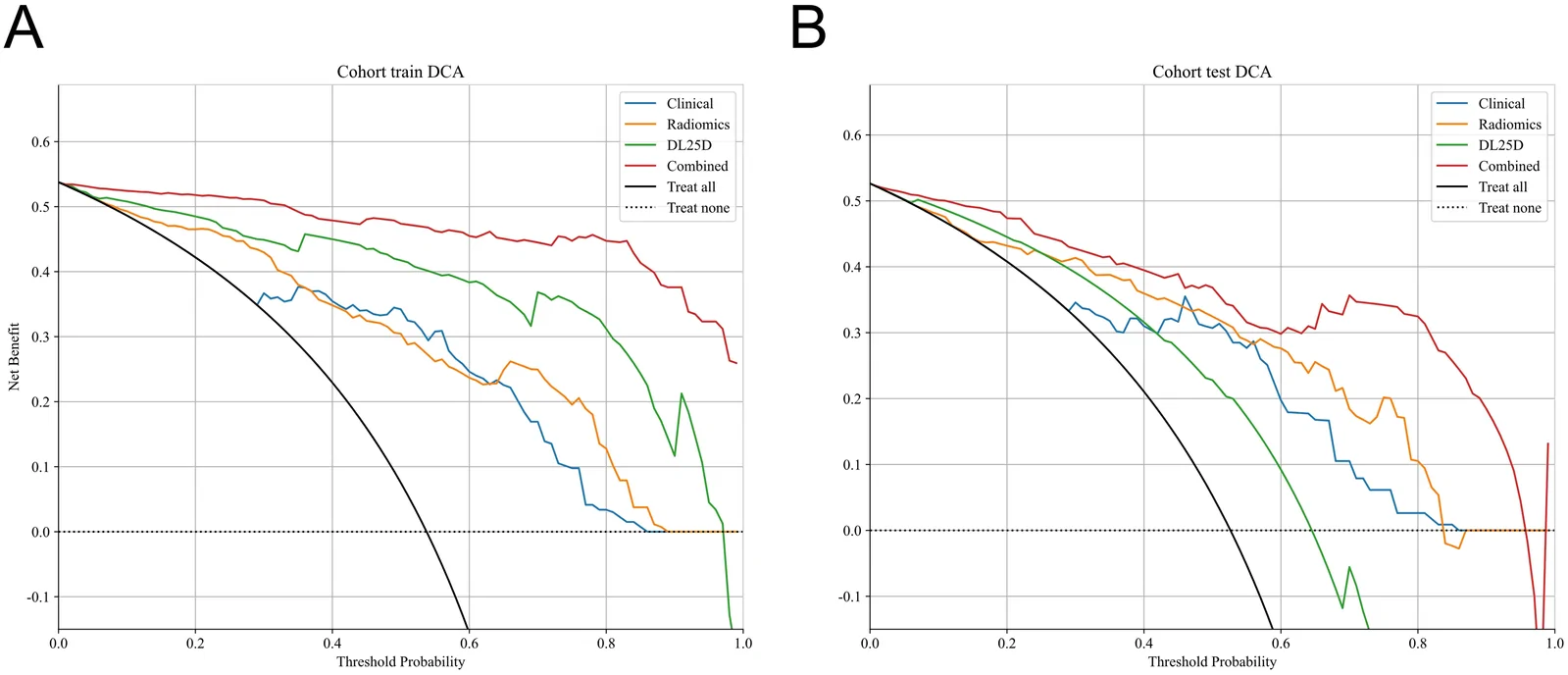
Model clinical value assessment ratio (**(A)** DCA curve of training cohort; **(B)** DCA curve of testing cohort).

## 4 Discussion

TM is a dimorphic fungus that exhibits a mycelial form at 25 °C and transforms into a yeast form at 37 °C or within host tissues. It has a distinct geographic distribution and is endemic mainly in Southeast Asian countries and southern China, particularly Guangxi, Guangdong, and Yunnan provinces (Morris et al., 2024; Zhou et al., 2025). TM infection is usually acquired through inhalation or inoculation through damaged skin. Due to its tropism for the mononuclear phagocyte system, dissemination can occur after invasion, and the lungs are among the most commonly involved organs (Brown et al., 2025). Concurrently, MTB infection remains high prevalent in patients with HIV/AIDS. Coinfection with HIV and MTB not only accelerates disease progression but also complicates the diagnosis of PTB and increases the risk of treatment failure (Yang et al., 2022). Differentiating TSM from PTB in patients with HIV/AIDS is challenging at multiple levels. Clinically, both diseases may present with non-specific manifestations such as prolonged fever, cough, expectoration, weight loss, and fatigue (Qiu et al., 2025; Zhao et al., 2025). Radiologically, there CT findings overlap substantially, including nodules, consolidation, and cavitary lesions, making visual differentiation difficult. Traditional diagnostic methods also have significant limitations: TM culture is time-consuming (5–14 days) and has limited sensitivity; the G-test lacks sufficient specificity; and sputum smear microscopy for PTB is associated with a relatively high false-negative rate. Consequently, neither conventional imaging nor etiologic or pathologic methods alone are always sufficient for accurate differentiation between the two conditions.

In this study, multivariate regression analysis identified CD4+ T lymphocyte count, platelet count, hemoglobin level, fever, diabetes, and hypertension as independent predictors for differentiating TSM from PTB. The predictive value of CD4+ T lymphocyte count has a clear immunological basis. Previous studies have suggested that tuberculosis tends to occur at relatively higher CD4+ T lymphocyte counts (200–500 cells/μL), whereas most fungal opportunistic infections occur when CD4+ T lymphocyte counts fall below 200 cells/μL (Rangwala et al., 2012; Mazumder et al., 2025). In this study, CD4+ T lymphocyte counts were lower in the TM group than that in the PTB group, which is consistent with this pattern. The independent associations of platelets count and hemoglobin level may reflect the specific tropism of TM for the mononuclear phagocyte system. Following TM infection, fungal proliferation in organs rich in mononuclear phagocytes, such as the bone marrow, ay suppress hematopoietic function and contribute to reductions in platelet count and hemoglobin. This nterpretation is in line with previous findings reported by Qiu et al., who also identified platelet count as a useful variable for differentiating TSM from PTB (Qiu et al., 2025). It is also noteworthy that diabetes and hypertension emerged as independent predictors. From a pathophysiologic perspective, diabetes is associated with impaired innate immune function, particularly reduced neutrophil chemotaxis, which may compromise clearance of MTB (Cheng et al., 2017). While hypertension itself does not directly alter immune status, it may reflect broader differences in baseline health statuss, healthcare access, or comorbidity burden. These findings suggest that, in patients with HIV/AIDS, non-HIV-related comorbidities also influence the spectrum of opportunistic infections, and should be considered in differential diagnosis.

With respect to image analysis, the 2.5D approach offers a practical compromise between 2D and 3D methods. Although 3D DL models can completely capture complete spatial information, they are associated with greater computational cost, higher hardware requirements, and an increased risk of overfitting when the sample size is limited. In contrast, 2D models are computationally efficient and easier to train, but they do not fully exploit inter-slice spatial context and may therefore provide less complete lesion characterization (Zhang et al., 2022). The 2.5D approach uses adjacent slices centered on the largest lesion cross-section to approximate 3D structural information. In this way, it retains much of the efficiency of 2D modeling while partially compensating for the lack of spatial context, without incurring the full technical burden of 3D models (Zhang et al., 2022; Yoo et al., 2023). In this study, the 2.5D DL framework used the slice with the largest lesion area together with adjacent slices at intervals of ±1, ± 2, and ±4 (seven slices total, comprising the central slice plus six neighboring slices), and patient-level predictions were aggregated using MIL. Among the slice-level models, ResNet18 achieved the best performance in the test set, with an AUC of 0.865. By contrast, although ResNet50 achieved a higher AUC in the training set, its performance declined substantially in the test set, suggesting overfitting. This observation is broadly consistent with a recent systematic review comparing 2D, 2.5D, and 3D networks, in which 2.5D models frequently achieved the best overall balance between performance and feasibility (Li et al., 2026).

The core finding of this study was the superior performance of the multimodal fusion model. In the test set, the combined model achieved an AUC of 0.931, representing an increase of 0.046 over the best single-modality model (DL25D, AUC = 0.885), and specificity improved from 0.926 to 0.981. This improvement is clinical meaningful. The high specificity (0.981) ensures that PTB patients (negative class) are protected from misclassification as TSM, thereby avoiding unnecessary antifungal therapy and its associated toxicities. However, the moderate sensitivity (0.717) indicates that 17 TSM cases were missed, highlighting the need for clinical vigilance when the model predicts PTB but clinical suspicion for TSM remains high. Although the AUC improvement of the combined model over DL25D did not reach statistical significance (P = 0.116), the substantial gain in specificity (0.981 vs. 0.926) is clinically meaningful, particularly in resource-limited settings where unnecessary antifungal therapy should be minimized. The combined model achieved substantially higher specificity (0.981) compared to DL25D (0.926), but at the cost of reduced sensitivity (0.717 vs. 0.667). This trade-off reflects the shift in the decision threshold toward reducing false positives, which is clinically advantageous in resource-limited settings where unnecessary antifungal therapy should be minimized. However, the moderate sensitivity indicates that some TSM cases were missed, highlighting the need for clinical vigilance when the model predicts PTB but suspicion for TSM remains high. Future work should explore threshold optimization to balance these competing risks based on clinical context and resource availability. The advantage of multimodal fusion likely reflects the complementary nature of the different data types. Clinical variables capture the host background, including the degree of immunosuppression, hematopoietic status, and comorbidity profile. Radiomic features quantify intralesional heterogeneity through gray-level and texture-based descriptors. The 2.5D DL features characterize lesion structure, margins, and spatial context through end-to-end representation learning. Together, these modalities provide a more comprehensive description of disease than any single modality alone. This interpretation is consistent with broader work on multimodal image fusion, which suggests that feature-level fusion can integrate complementary information and improve predictive performance (Zubair et al., 2025). Our Grad-CAM analysis extends beyond ‘black-box’ visualization by grounding model attention in established pulmonary pathology. The activation maps for two representative true-positive cases (one PTB, one TSM) demonstrate that the model focuses on regions containing the pulmonary lesions rather than background anatomy. In the PTB case, activation is concentrated within and around the cavitary lesion; in the TSM case, activation overlies the nodular infiltrates. These visualizations suggest that the model attends to clinically relevant image regions, though they do not demonstrate recognition of specific pathological features such as cavity-wall irregularity or satellite nodules. In the sex subgroup analysis, the combined model achieved an AUC of 0.926 (95% CI: 0.874–0.978) in males and 0.941 (95% CI: 0.894–0.988) in females, with no statistically significant difference (P = 0.672). However, with only 25 women in the test set (8 TSM, 17 PTB), this analysis is severely underpowered to detect moderate differences in model performance. Therefore, while we found no evidence of sex-related performance disparity, we cannot conclude that the model is truly unaffected by sex. Larger prospective studies with balanced sex representation are required to definitively establish the model’s generalizability across genders.

Several limitations should also be acknowledged. First, this is a single-center retrospective study with internal validation only. While we employed rigorous nested cross-validation to minimize overfitting, the generalizability of our model to other geographic regions, different HIV/AIDS populations, or alternative CT protocols remains uncertain. Prospective external validation in independent cohorts is essential before clinical deployment. Second, while our model demonstrates excellent discrimination, this does not directly translate to reduced diagnostic delay or improved patient outcomes. Future studies should assess whether integration of this model into clinical workflows actually improves time-to-diagnosis or patient survival. It is important to emphasize that this study specifically addressed the binary differentiation between TSM and PTB in HIV/AIDS patients without coinfections or other opportunistic pulmonary infections. While this design ensured a homogeneous cohort for algorithm development, our conclusions cannot be extrapolated to the broader clinical scenario of HIV/AIDS patients with polymicrobial infections, mixed infections, or other pulmonary complications. Future studies should expand the differential diagnosis framework to include a wider spectrum of pulmonary pathogens and coinfection states to enhance clinical utility. Third, although manual ROI delineation by two experienced radiologists achieved good inter-observer reproducibility (ICC > 0.8), it is time-consuming and subject to variability, hindering real-world clinical deployment, especially for 3D VOI construction on consecutive slices. To overcome this, future work should integrate an automated segmentation network (e.g., 3D U-Net or nnU-Net) before the classification step. A well-validated fully automated approach would reduce manual effort, eliminate observer bias, and facilitate integration into routine practice.

## 5 Conclusions

This study developed and validated a CT-based multimodal 2.5D DL model for differentiating TSM from PTB in HIV/AIDS patients. In the test set, the multimodal fusion model integrating clinical, radiomics, and 2.5D DL features achieved an AUC of 0.931 and a specificity of 0.981, outperforming the single-modality models. These findings support the complementary value of host-related clinical information, radiomics-derived lesion heterogeneity, and DL-based spatial representations, and suggest a potentially useful approach for the intelligent differential diagnosis of complex infectious lung diseases.

## Data Availability

The original contributions presented in the study are included in the article/**Supplementary Material**. Further inquiries can be directed to the corresponding authors.
