# Supplementary material for: Differential diagnosis of pulmonary Talaromyces marneffei infection and pulmonary tuberculosis in HIV/AIDS patients using a CT-based multimodal 2.5D deep learning approach
Source: Front Cell Infect Microbiol. 2026 Oct 23;16:1927286. doi: 10.3389/fcimb.2026.1927286 (PMC13739602; doi:10.3389/fcimb.2026.1927286)
Supplement: Supplementary file 1 [file Supplementaryfile1.docx]

**Supplementary Material**

**1A. Details Multi-Instance Learning-Based Feature Fusion**

In our study, we implemented two multi-instance learning fusion techniques. Using 2.5D deep learning models, we created Predict Likelihood Histograms (PLH) that map out the predictive probabilities and labels for each slice, offering a probabilistic summary of the prediction landscape. We also applied a Bag of Words (BoW) approach, segmenting each image into slices and extracting data to compile seven predictive results per sample, using the Term Frequency-Inverse Document Frequency (TF-IDF) method for analysis. Additionally, we enhanced our model by integrating PLH and BoW features with radiomic data, leveraging diverse data sources to improve the representational power and accuracy of our classification tasks.

Our multi-instance learning approach aimed to enhance predictive accuracy by integrating various data points from a single sample into a comprehensive feature set, involving:

1. **Slice Prediction**: Each slice was analyzed using the deep learning model to derive probabilities and labels, denoted as $Slice_{prob}$ and $Slice_{pred}$, retained to two decimal places.
2. **Multi Instance Learning Feature Aggregation**:
   - **Histogram Feature Aggregation**:
     - Distinct numbers were treated as "bins" to count occurrences across types.
     - Frequencies of $Slice_{prob}$ and $Slice_{pred}$ in each bin were tallied and normalized using min-max normalization, resulting in $Histo_{prob}$ and $Histo_{pred}$.
   - **Bag of Words (BoW) Feature Aggregation**:
     - A dictionary was constructed from unique elements in $Slice_{prob}$ and $Slice_{pred}$.
     - Each slice was represented as a vector noting the frequency of each dictionary element, with a TF-IDF transformation applied to emphasize informative features.
     - This resulted in a BoW feature representation for each slice, encapsulating both the presence and significance of features.
3. **Feature Early Fusion**: We integrated $Histo_{prob}$, $Histo_{pred}$, $Bow_{prob}$, and $Bow_{pred}$ using a feature concatenation method ($\oplus$), combining these into a single comprehensive feature vector:

$$feature_{fusion}=Histo_{prob}\oplus Histo_{pred}\oplus Bow_{prob}\oplus Bow_{pred}$$

For the aggregated multi-instance learning features, we utilized dimensionality reduction techniques such as t-tests, correlation coefficients, and Lasso regularization to refine our feature set. These features were modeled using popular machine learning algorithms including Logistic Regression and ExtraTrees. To address sample imbalance, we employed the SMOTE method during the training process. To ensure model robustness, we applied 5-fold cross-validation within the training dataset and optimized hyperparameters via Grid-Search.

**1B. Details Different Ensemble Fusion Method**

Leveraging the concept of ensemble methods, we combined the predicted probabilities from different cross-sectional images of the same patient to enhance predictive performance and robustness. Specifically, we implemented two distinct approaches to perform this ensemble fusion: using the maximum and the average values of the predictions.

The first approach involves taking the maximum predicted probability among all slices for a given patient. This method tends to highlight the most abnormal features detected across slices, which might indicate the presence of pathological findings more assertively. The mathematical representation for the maximum ensemble method is as follows:

$$P_{\text{max}}=max\left( P_{+1},P_{+2},P_{+4},P_{max\_roi},P_{-1},P_{-2},P_{-4} \right)$$

The second approach averages the predicted probabilities across all slices. This method provides a more balanced view that incorporates contributions from all slices, potentially smoothing out any anomalies that might appear in a single slice. The formula for calculating the average predicted probability is:

$$P_{\text{mean}}=\frac{1}{n}\sum\left( P_{+1},P_{+2},P_{+4},P_{max\_roi},P_{-1},P_{-2},P_{-4} \right)$$

where $P_{\text{max}}$ is the maximum probability, where$P_{\text{mean}}$ represents the average probability and $P_{+1},P_{+2},P_{+4},P_{max\_roi},P_{-1},P_{-2},P_{-4}$are the predicted probabilities for each slice.

For each slice, the CNN produced a probability output and a discrete class prediction. Probability values were rounded to two decimal places. In binary tasks, the class prediction was coded as 0 or 1; in multi-class tasks, it was retained as the predicted class index. Fixed histogram bins were defined using only the training folds and were then applied unchanged to validation and test data. For probability features, 101 bins were used from 0.00 to 1.00 with a step of 0.01; for predicted-label features, bins corresponded to the unique class labels observed in the training folds. For each patient, the seven slices were counted across these bins, and the resulting frequencies were min–max normalized using the minimum and maximum values from the training folds to generate $Histo_{prob}$and $Histo_{pred}$. If a feature was constant in the training folds, its normalized value was set to 0.

For the BoW representation, dictionaries were constructed exclusively from the training folds. The probability dictionary contained the 101 unique rounded probability values, and the label dictionary contained the unique predicted class labels. Each patient’s seven slices were encoded as count vectors over the corresponding dictionary, and the counts were summed to obtain patient-level term frequencies. The term frequency was defined as $TF\left( t , d \right)=count\left( t , d \right)/7$, and the inverse document frequency was defined as $IDF\left( t \right)=\ln\left( \left( 1+N \right)/\left( 1+df\left( t \right) \right) \right)+1$, where $N$was the number of patients in the training fold and $df\left( t \right)$was the number of patients containing term $t$. The TF–IDF weight was $TF\times IDF$, and the resulting patient-level vector was L2-normalized, yielding $Bow_{prob}$and $Bow_{pred}$. Terms absent from the training dictionary were ignored in validation and test data, and the dictionary was not updated using validation or test data. The final DL25D representation was $feature_{fusion}=Histo_{prob}\oplus Histo_{pred}\oplus Bow_{prob}\oplus Bow_{pred}$.

The patient-level DL25D classifier was a support vector machine with a linear kernel. Hyperparameters were optimized by grid search and five-fold cross-validation performed exclusively within the outer training folds. The regularization parameter $C$was searched over $\left\{ 0.001 , 0.01 , 0.1 , 1 , 10 , 100 \right\}$, and class_weight was searched over $\left\{ None,^{'}balanced^{'} \right\}$. SMOTE was applied only to the training folds and never to validation folds or the locked test set. The final combined model was constructed as $feature_{combined}=feature_{fusion}\oplus radiomics_{selected}\oplus clinical_{selected}$and used the same linear-kernel SVM and the same hyperparameter search strategy. All preprocessing, probability rounding rules, histogram bins, BoW dictionaries, TF–IDF parameters, SMOTE, and classifier hyperparameters were fitted on training folds only, ensuring that the validation and test sets remained untouched until final evaluation.

The DL25D signature refers exclusively to the patient-level representation obtained by applying MIL to the 2.5D CNN slice-level predictions. It consists only of the four MIL feature groups, namely $Histo_{prob}$, $Histo_{pred}$, $Bow_{prob}$, and $Bow_{pred}$, and is denoted as $feature_{fusion}$. It does not contain any radiomics or clinical variables. Radiomics features are introduced only in the final Combined model, in which the DL25D MIL representation is concatenated with the selected radiomics features and selected clinical features:

$$feature_{combined}=feature_{fusion}\oplus radiomics_{selected}\oplus clinical_{selected}.$$

Detailed comparisons between these two ensemble methods are available, offering insights into their respective advantages and limitations in the context of medical image analysis. These comparative analyses help in selecting the most appropriate ensemble strategy based on specific clinical and diagnostic requirements.

| Method | AUC | 95% CI | Sensitivity | Specificity | PPV | NPV | Cohort |
| --- | --- | --- | --- | --- | --- | --- | --- |
| mean | 0.985 | 0.9744-0.9965 | 0.944 | 0.959 | 0.964 | 0.937 | train |
| mean | 0.873 | 0.8124-0.9339 | 0.900 | 0.648 | 0.740 | 0.854 | test |
| max | 0.958 | 0.9366-0.9802 | 0.902 | 0.919 | 0.928 | 0.890 | train |
| max | 0.880 | 0.8216-0.9392 | 0.717 | 0.907 | 0.896 | 0.742 | test |

To prevent data leakage between CNN training and MIL classifier construction, we employed a patient-level nested cross-validation strategy strictly within the training cohort. Specifically, the training set was first partitioned into five outer folds at the patient level. For each outer training fold, we further divided it into inner training and inner validation folds. The slice-level CNN model was trained exclusively on the inner training folds, and subsequently generated out-of-fold predictions for patients in the inner validation folds. These out-of-fold slice-level probabilities and labels were used to construct MIL features (PLH and BoW) and to train the patient-level MIL classifier, while the outer validation fold was used to evaluate the generalization performance of the MIL classifier. This procedure was repeated five times to obtain stable cross-validated performance estimates.

For final model deployment, the CNN was retrained on the entire training set to generate slice-level predictions for the locked test set. Concurrently, the MIL classifier was trained using out-of-fold predictions derived from all training set patients (generated via the nested cross-validation procedure described above), and then applied to predict patient-level outcomes for the test set. This ensured that the slice-level predictions used to train the MIL classifier never originated from patients whose data were used to train the CNN, thereby eliminating information leakage and maintaining the integrity of model evaluation.

**Supplementary Table 1. Baseline characteristic of TSM and PTB cohorts.**

| Feature Name | ALL (N=380) | TSM(N=177) | PTB(N=203) | P value |
| --- | --- | --- | --- | --- |
| gender |  |  |  |  |
| 0 | 84 (22.11%) | 43 (24.29%) | 41 (20.20%) | 0.403 |
| 1 | 296 (77.89%) | 134 (75.71%) | 162 (79.80%) |  |
| age | 46.82 (14.60) | 43.38 (14.11) | 49.82 (14.39) | <0.001 |
| hypertension | 22 (5.79%) | 0 (0.00%) | 22 (10.84%) | <0.001 |
| diabetes | 15 (3.95%) | 1 (0.56%) | 14 (6.90%) | 0.004 |
| smoke | 100 (26.32%) | 40 (22.60%) | 60 (29.56%) | 0.156 |
| drink | 78 (20.53%) | 33 (18.64%) | 45 (22.17%) | 0.471 |
| fever | 191 (50.26%) | 116 (65.54%) | 75 (36.95%) | <0.001 |
| CD3 | 450.00 (216.00±877.00) | 291.00 (152.00±492.00) | 704.00 (338.50±1083.50) | <0.001 |
| CD4 | 48.00 (14.00±183.50) | 17.00 (7.00±48.00) | 127.00 (39.50±342.50) | <0.001 |
| CD8 | 344.00 (177.25±597.75) | 254.00 (130.00±432.00) | 471.00 (249.00±668.50) | <0.001 |
| CD4/CD8 | 0.13 (0.06±0.40) | 0.08 (0.04±0.15) | 0.27 (0.10±0.63) | <0.001 |
| WBC | 4.79 (3.13±6.89) | 4.00 (2.70±6.00) | 5.40 (3.60±7.40) | <0.001 |
| RBC | 3.39 (2.75±3.98) | 3.16 (2.55±3.75) | 3.66 (2.90±4.28) | <0.001 |
| hemoglobin | 93.00 (71.00±111.25) | 81.00 (66.00±101.00) | 102.00 (85.00±120.00) | <0.001 |
| platelet | 189.00 (116.00±268.75) | 137.00 (68.00±221.00) | 219.00 (171.50±295.00) | <0.001 |
| neutrophil | 3.20 (1.92±5.20) | 3.00 (1.78±4.55) | 3.30 (2.12±5.58) | 0.017 |
| lymphocyte | 0.70 (0.40±1.30) | 0.58 (0.30±0.84) | 0.97 (0.60±1.50) | <0.001 |
| monocyte | 0.38 (0.20±0.50) | 0.28 (0.18±0.47) | 0.40 (0.30±0.51) | <0.001 |
| eosinophils | 0.06 (0.01±0.14) | 0.04 (0.01±0.13) | 0.07 (0.01±0.15) | 0.113 |
| basophilia | 0.02 (0.01±0.03) | 0.02 (0.01±0.04) | 0.02 (0.01±0.03) | 0.056 |
| CRP | 23.10 (5.00±63.83) | 31.60 (9.30±72.70) | 15.00 (5.00±51.75) | <0.001 |

**Supplementary Table 2.** Baseline characteristic of test and train cohorts.

| Feature Name | ALL (N=380) | test (N=114) | train (N=266) | P value |
| --- | --- | --- | --- | --- |
| age | 46.82 ±14.60 | 45.63±15.57 | 47.33±14.16 | 0.319 |
| CD3 | 450.00 (216.00, 877.00) | 453.50 (232.50, 836.75) | 450.00 (211.50, 880.00) | 0.936 |
| CD4 | 48.00 (14.00, 183.50) | 42.00 (13.00, 154.25) | 48.50 (14.25, 187.75) | 0.393 |
| CD8 | 344.00 (177.25, 597.75) | 331.00 (185.00, 597.00) | 345.50 (173.50, 598.50) | 0.735 |
| CD4/CD8 | 0.13 (0.06, 0.40) | 0.13 (0.05, 0.37) | 0.13 (0.06, 0.41) | 0.449 |
| WBC | 4.79 (3.13, 6.89) | 4.60 (3.12, 6.30) | 4.80 (3.14, 7.16) | 0.188 |
| RBC | 3.39 (2.75, 3.98) | 3.24 (2.74, 3.98) | 3.42 (2.76, 3.98) | 0.638 |
| hemoglobin | 93.00 (71.00, 111.25) | 93.50 (75.00, 112.00) | 92.50 (70.00, 110.75) | 0.699 |
| platelet | 189.00 (116.00, 268.75) | 201.50 (118.75, 271.00) | 186.00 (116.00, 267.75) | 0.892 |
| neutrophil | 3.20 (1.92, 5.20) | 3.10 (1.94, 4.68) | 3.25 (1.91, 5.30) | 0.561 |
| lymphocyte | 0.70 (0.40, 1.30) | 0.70 (0.40, 1.00) | 0.70 (0.40, 1.37) | 0.191 |
| monocyte | 0.38 (0.20, 0.50) | 0.30 (0.20, 0.50) | 0.40 (0.20, 0.50) | 0.241 |
| eosinophils | 0.06 (0.01, 0.14) | 0.05 (0.01, 0.10) | 0.06 (0.01, 0.15) | 0.055 |
| basophilia | 0.02 (0.01, 0.03) | 0.02 (0.01, 0.03) | 0.02 (0.01, 0.04) | 0.120 |
| CRP | 23.10 (5.00, 63.83) | 22.50 (6.00, 62.70) | 23.30 (5.00, 64.57) | 0.784 |
| gender |  |  |  | 1.000 |
| 0 | 84(22.11) | 25(21.93) | 59(22.18) |  |
| 1 | 296(77.89) | 89(78.07) | 207(77.82) |  |
| hypertension |  |  |  | 1.000 |
| 0 | 358(94.21) | 107(93.86) | 251(94.36) |  |
| 1 | 22(5.79) | 7(6.14) | 15(5.64) |  |
| diabetes |  |  |  | 0.565 |
| 0 | 365(96.05) | 108(94.74) | 257(96.62) |  |
| 1 | 15(3.95) | 6(5.26) | 9(3.38) |  |
| smoke |  |  |  | 0.525 |
| 0 | 280(73.68) | 87(76.32) | 193(72.56) |  |
| 1 | 100(26.32) | 27(23.68) | 73(27.44) |  |
| drink |  |  |  | 0.561 |
| 0 | 302(79.47) | 88(77.19) | 214(80.45) |  |
| 1 | 78(20.53) | 26(22.81) | 52(19.55) |  |
| fever |  |  |  | 1.000 |
| 0 | 189(49.74) | 57(50.00) | 132(49.62) |  |
| 1 | 191(50.26) | 57(50.00) | 134(50.38) |  |

**Supplementary Table 3.** Multivariable logistic regression analysis of clinical features in the training cohort (N=266).

| Feature Name | OR_UNI | 95% CI_UNI | P_UNI | OR_MULTI | 95% CI_MULTI | P_MULTI |
| --- | --- | --- | --- | --- | --- | --- |
| basophilia | 0.304 | 0.146-0.633 | <0.05 | 0.469 | 0.229-0.963 | 0.052 |
| fever | 0.774 | 0.702-0.854 | <0.05 | 0.892 | 0.813-0.978 | <0.05 |
| eosinophils | 0.955 | 0.776-1.176 | 0.716 |  |  |  |
| CD4/CD8 | 0.998 | 0.994-1.002 | 0.391 |  |  |  |
| CRP | 0.999 | 0.998-1.000 | <0.05 | 1.0 | 0.999-1.001 | 0.499 |
| CD3 | 1.000 | 1.000-1.000 | <0.05 | 1.0 | 0.999-1.000 | 0.122 |
| CD8 | 1.000 | 1.000-1.000 | <0.05 | 1.0 | 1.000-1.001 | 0.167 |
| CD4 | 1.001 | 1.001-1.001 | <0.05 | 1.001 | 1.000-1.001 | <0.05 |
| RBC | 1.002 | 0.999-1.004 | 0.197 |  |  |  |
| platelet | 1.002 | 1.001-1.002 | <0.05 | 1.001 | 1.001-1.002 | <0.05 |
| hemoglobin | 1.006 | 1.005-1.008 | <0.05 | 1.004 | 1.002-1.006 | <0.05 |
| age | 1.006 | 1.002-1.009 | <0.05 | 1.001 | 0.998-1.004 | 0.627 |
| neutrophil | 1.014 | 1.000-1.029 | 0.112 |  |  |  |
| WBC | 1.017 | 1.003-1.030 | <0.05 | 0.997 | 0.982-1.012 | 0.771 |
| drink | 1.076 | 0.946-1.221 | 0.347 |  |  |  |
| gender | 1.084 | 0.960-1.225 | 0.273 |  |  |  |
| lymphocyte | 1.089 | 1.027-1.155 | <0.05 | 1.017 | 0.946-1.094 | 0.7 |
| Heart Disease | 1.101 | 0.923-1.314 | 0.37 |  |  |  |
| smoke | 1.180 | 1.054-1.320 | <0.05 | 1.093 | 0.993-1.203 | 0.127 |
| monocyte | 1.503 | 1.224-1.846 | <0.05 | 0.939 | 0.755-1.168 | 0.634 |
| diabetes | 1.614 | 1.225-2.125 | <0.05 | 1.421 | 1.115-1.809 | <0.05 |
| hypertension | 1.632 | 1.318-2.022 | <0.05 | 1.283 | 1.058-1.557 | <0.05 |

**Supplementary Figure 1.** Workflow for the feature selection process in radiomics. (A. Binomial deviance (log-loss) across 10-fold cross-validation, with error bars representing standard error; B. Coefficient paths across the regularization parameter lambda; C. Coefficients of the selected radiomics features.)
